# The Role of Histone Methylation and H2A.Z Occupancy during Rapid Activation of Ethylene Responsive Genes

**DOI:** 10.1371/journal.pone.0028224

**Published:** 2011-11-28

**Authors:** Yongfeng Hu, Yuan Shen, Natalia Conde e Silva, Dao-Xiu Zhou

**Affiliations:** Institut de Biologie des Plantes, Université Paris-Sud, Orsay, France; French National Centre for Scientific Research, France

## Abstract

Ethylene signaling pathway leads to rapid gene activation by two hierarchies of transcription factors with EIN3/EIL proteins as primary ones and ERF proteins as secondary ones. The role of chromatin modifications during the rapid gene activation is not known. In this work we studied trimethylated histone H3 lysine 4 (H3K4me3) and lysine 27 (H3K27me3), two opposite histone methylation marks for gene activity, during the induction course of three ethylene-responsive genes (*ERF1*, *AtERF14* and *ChiB*). We found that the three genes displayed different histone modification profiles before induction. After induction, H3K4me3 was increased in the 5′ region and the gene body of *ERF1*, while H3K27me3 was decreased in the promoter of *AtERF14*. But the modification changes were later than the gene activation. Analysis of other rapidly inducible *ERF* genes confirmed the observation. In addition, histone H2A.Z occupancy on the three genes and the association of the H3K27me3-binding protein LHP1 with *AtERF14* and *ChiB* were not affected by the inductive signal. However, the mutation of genes encoding H2A.Z and LHP1 attenuated and enhanced respectively the induction of target genes and altered H3K4me3. These results indicate that the induction of ethylene-responsive genes does not require immediate modulation of H3K4me3 and H3K27me3 and dissociation of LHP1 and H2A.Z from the targets, and suggest that the chromatin structure of the genes before induction is committed for transcriptional activation and that H3K4me3 is not required for ethylene-responsive gene activation, but may serve as a mark for gene activity.

## Introduction

In addition to transcription factors chromatin structure plays an important role in the regulation of gene expression. The basic unit of chromatin is nucleosome that is formed by histone octamer containing two copies of H3, H4, H2A and H2B wrapped around by 147 base pairs of DNA. Chromatin structure change includes histone modifications and DNA methylation, histone variant deposition and chromatin remodeling. Histone modifications, especially H3K4 trimethylation and H3K27 trimethylation, have been largely reported to be tightly associated with gene transcription activity [1], [2]. H3K4me3 is associated with highly expressed and/or housekeeping genes whereas H3K27me3 marks under-expressed and/or repressed tissue-specific genes [1], [2]. Both modification marks could be recognized by different chromatin factors through specific protein domains. For example, the Plant Homeodomain (PHD) of ING2 (Inhibitor of Growth 2) can bind to H3K4me3 and the chromodomain of Polycomb proteins in animal cells and LIKE HETEROCHROMATIN PROTEIN1 (LHP1) in Arabidopsis can bind to H3K27me3 [3], [4]. The recognitions may serve as a mechanism by which histone modifications regulate gene expression. Histone variant H2A.Z is another important regulator of gene expression which is deposited into nucleosome by SWR complex. Recent analysis in various species has revealed that activation of H2A.Z-regulated genes was accompanied by eviction of H2A.Z or replacement of H2A.Z with H2A by INO80 complex [5], [6], [7]. Other studies have suggested that H2A.Z may act as an epigenetic mark to promote gene reactivation [8], [9].

In plants, H3K4me3 and the H3K27me3/LHP1 module have been shown to mediate developmental genes expression such as *FLC* (*FLOWERING LOCUS C*), *AG* (*AGAMOUS*), *FUS3* (*FUSCA 3*) and *FT* (*FLOWERING LOCUS T*) [10], [11], [12], [13]. However, how these modifications affect rapidly induced gene activation was not clear. Ethylene is a plant hormone participating in different processes including germination, flower and leaf senescence, fruit ripening, leaf abscission, root nodulation, programmed cell death, and response to stress and pathogen attack. Genetic and molecular analyses have revealed a response pathway from perception to a series of MAP kinase and finally transduced to two hierarchies of transcription regulation [14]. The primary transcription regulation is that transcription factors EIN3 (ETHYLENE-INSENSITIVE3)/EIL1 (ETHYLENE-INSENSITIVE3-LIKE 1) directly bind to EREBP (ethylene-responsive element binding protein) genes such as *ERF1* (*ETHYLENE RESPONSE FACTOR 1*) to activate their expression. Subsequently EREBP proteins activate downstream effecter genes (e.g. *ChiB*, *basic chitinase* and *PDF1.2*, *Plant Defensin 1.2*). However, it was not known whether the rapid activation of ethylene-responsive genes involves change of chromatin structure. Here, we chose *ERF1* and *AtERF14* (*Arabidopsis thaliana Ethylene-responsive element binding factor 14*) as well as 5 other *ERF* genes as primary and *ChiB* as secondary regulation targets to analyze whether chromatin structures of these target genes changed during rapid induction by ethylene. We used 1-aminocyclopropane-1-carboxylic acid (ACC) which is converted to ethylene by 1-aminocyclopropane-1-carboxylic acid oxidase (ACO) in plants to treat 12 day-old seedlings. Increase of H3K4me3 and decrease of H3K27me3 were observed during the treatment, but the changes of both marks were much later than the gene activation. H2A.Z occupancy and LHP1 binding did not respond to the treatment indicating that the gene induction by ethylene signaling did not require immediate change of the cognate chromatin structure. However, mutation of genes encoding H2A.Z and LHP1 affected the induction of ethylene-responsive genes, suggesting that the committed chromatin structure of these genes before induction is important for the transcriptional activation.

## Results

### Histone methylation profile and H2A.Z deposition over ethylene-responsive genes before induction

**Figure 1:**
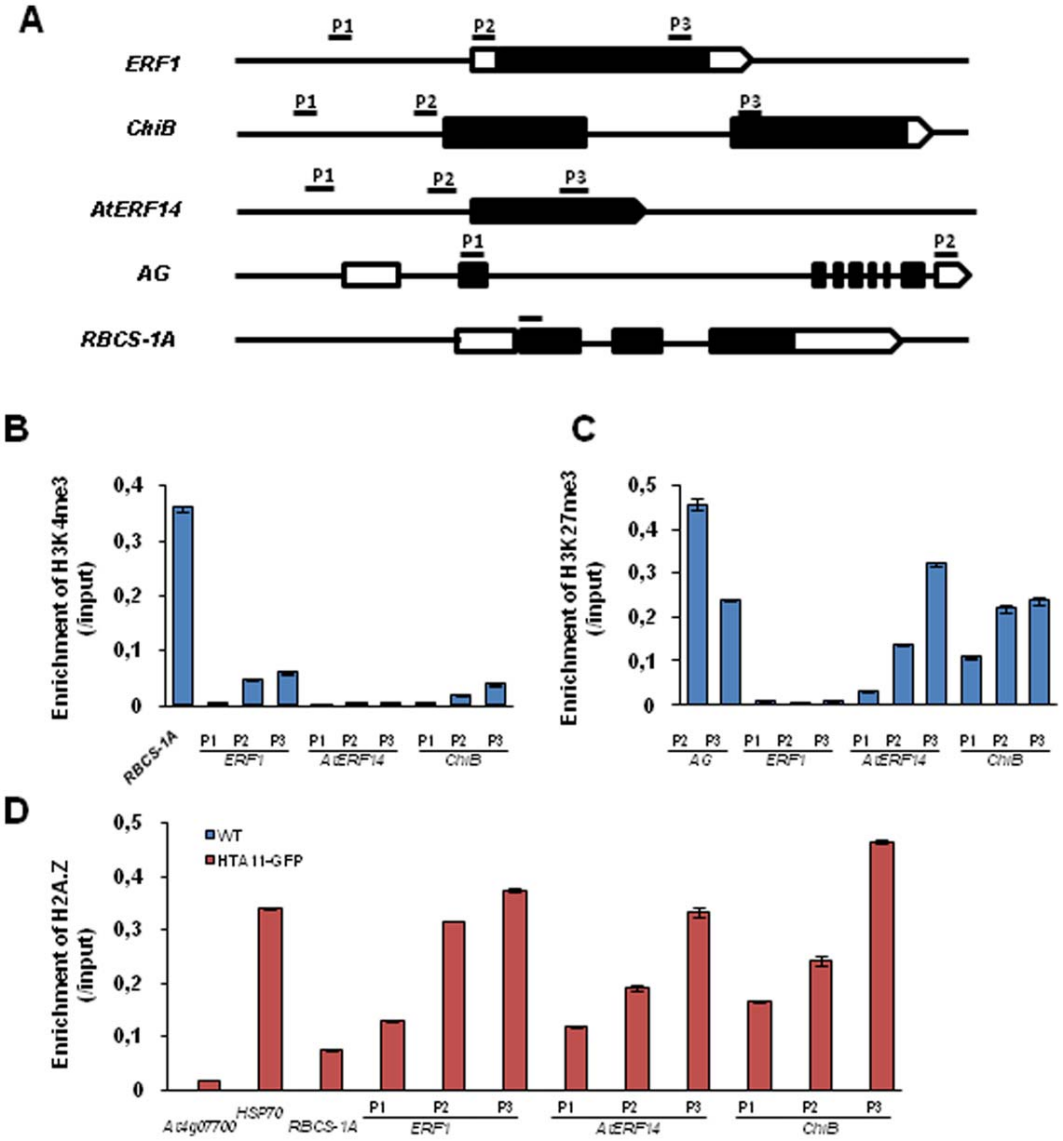
Chromatin status of *ERF1*, *AtERF14* and *ChiB* before ACC induction. (A) Diagrams of the gene structure of *ERF1*, *AtERF14*, *ChiB*, *AG*, and *RBCS1A*. The solid boxes indicate the coding regions, and the open boxes indicate untranslated regions (UTRs). The solid bars indicate the regions in which primers were designed for ChIP tests. (B) H3K4me3 on the three genes before induction. The relative enrichments were calculated by compared to input. RBCS was used as a positive control. The regions (P1, P2 and P3) shown in A represent promoter, 5′ region and gene body respectively. (C) H3K27me3 on the three genes before induction. AG was used as a positive control. (D) H2A.Z abundance on the three genes before induction. HSP70 was used as a positive control and At4g07700 was used as a negative control. Bars represent mean values +/−SD from three repeats.

To assess the chromatin structure of ethylene-responsive genes before induction we tested H3K4me3, H3K27me3 and H2A.Z deposition in the promoter, the 5′ region and the gene body of *ERF1*, *AtERF14* and *ChiB* (Fig. 1A). *RBCS-1A* (*RIBULOSE BISPHOSPHATE CARBOXYLASE SMALL CHAIN 1A*), *AG* and *HSP70* (*heat shock protein 70*) were used as positive controls respectively for H3K4me3, H3K27me3 and H2A.Z deposition [1], [5], [13]. The *At4g07700* locus was used as negative control for H2A.Z deposition [5]. Moderate levels of H3K4me3 were detected in the 5′ region and the gene body, but not the promoter, of *ERF1* and *ChiB* compared to that of *RBCS-1A* (Fig. 1B). In contrast, H3K4me3 was not detected over *AtERF14* (Fig. 1B). H3K27me3 was enriched in the 5′ region and the gene body of both *AtERF14* and *ChiB* but not in *ERF1* (Fig. 1C). Analysis of five additional *ERF* genes (*ORA59*, *TDR1*, *AtERF1*, *AtERF2* and *AtERF11*) revealed that *TDR1* displayed a high level of H3K27me3 but a low level of H3K4me3, while the other four genes showed a high level of H3K4me3 but a low level of H3K27me3 (Fig. S2). Similar to what found in *ERF1* and *ChiB*, H3K4me3 levels on the promoter of these genes were relatively low (Fig. S2). This analysis revealed that the ethylene-inducible genes displayed different histone modification profiles before induction.

To test whether H2A.Z was present in the chromatin of these genes, chromatin fragments isolated from H2A.Z-GFP transgenic plants were precipitated with GFP antibody. We found that H2A.Z was incorporated into chromatin over the three genes with highest levels in the gene bodies and lowest levels in the promoters (Fig. 1D). The presence of H2A.Z was detected also over the five additional *ERF* genes (Fig. S2).

### Histone methylation dynamics during induction of ethylene responsive genes

**Figure 2:**
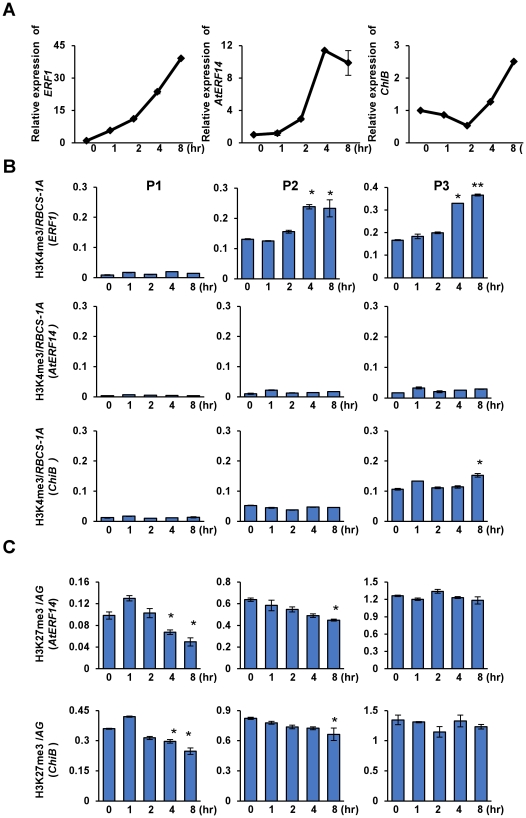
Expression and histone modification changes of three ethylene responsive genes during ACC induction. (A) Induction time course of the three genes by ACC. Twelve day-old seedlings were treated with 50 µM ACC and harvested at the indicated time points. Relative fold changes were determined by normalization with *ACTIN2* transcript levels. (B) H3K4me3 detected on the three genes during ACC induction. The relative enrichments were calculated by first comparing to input and then to the reference gene *RBCS-1A*. The three regions were analyzed for ChIP. (C) H3K27me3 detected on the three genes during ACC induction. The relative enrichments were calculated by first comparing to input and then to the reference gene *AGAMOUS* (*AG*). The three regions were analyzed for ChIP. Bars represent mean values +/−SD from three repeats. Significance of H3K4me3 and H3K27me3 differences between after ACC treatment (at different time points) and before treatment (0) was tested by two-tailed Student's t-test, * p<0.05, **p<0.005.

In order to study histone modification dynamics during gene activation, we chose five time points to monitor ACC induction time course of ethylene-responsive genes by quantitative RT-PCR. The induction of *ERF1* by ACC was early, which was 4 folds after 1 hour and elevated to 40 folds after 8 hours (Fig. 2A). For *AtERF14*, the expression began to increase after 2 hours and reached to 12 folds after 8 hours (Fig. 2A). The induction of *ChiB* was moderate, only 2 to 3 folds after 8 hours (Fig. 2A). *ORA59*, *AtERF1*, *AtERF2* and *AtERF11*were induced as early as *ERF1*, while the induction of *TDR1* was delayed (Fig. S2).

H3K4me3 and H3K27me3 are two opposite histone modification marks associated with gene transcription activity. However, it is still not clear whether the two modifications are involved in gene activation process. Therefore, we tested the levels of these modifications over ethylene responsive genes during the ACC induction process. For *ERF1*, H3K4me3 in the 5′ region and the gene body began to increase only after 4 hours, which was later than the initial increase of gene expression (Fig. 2B). Similarly, the increase of H3K4me3 over *ORA59*, *AtERF1*, *AtERF2* and *AtERF11* was also later than gene activation (Fig. S2). This suggested that the induction of *ERF1* did not require a concurrent increase of H3K4me3. The late increase might indicate that H3K4me3 served as a mark of elevated transcription activity of the genes. H3K4me3 remained undetectable over *AtERF14* and *TDR1*and did not change over *ChiB* during the induction (Fig. 2B) (Fig. S2). These results indicated that H3K4me3 was not necessary for the induction of *AtERF14* and *TDR1*. However, it was not clear whether the basal levels of H3K4me3 over *ERF1* and *ChiB* before induction was required for the induction of the genes.

Due to the low level of H3K27me3 over *ERF1* before induction we did not expect that there would be any change during the induction. So we tested H3K27me3 over *AtERF14* and *ChiB*. H3K27me3 was not much changed in the gene body of both genes during induction, but was decreased in the promoter of the genes, especially *AtERF14* (Fig. 2C). However the decrease of H3K27me3 was delayed compared to the gene induction, suggesting that rapid gene activation did not require or lead to immediate demethylation of H3K27me3 and that the presence of H3K27me3 did not prevent the induction process. Analysis of H3K27me3 over *ORA59*, *TDR1*, *AtERF1*, *AtERF2* and *AtERF11* confirmed the results (Fig. S2).

### Negative function of LHP1 on the induction of *AtERF14* and *ChiB*

**Figure 3:**
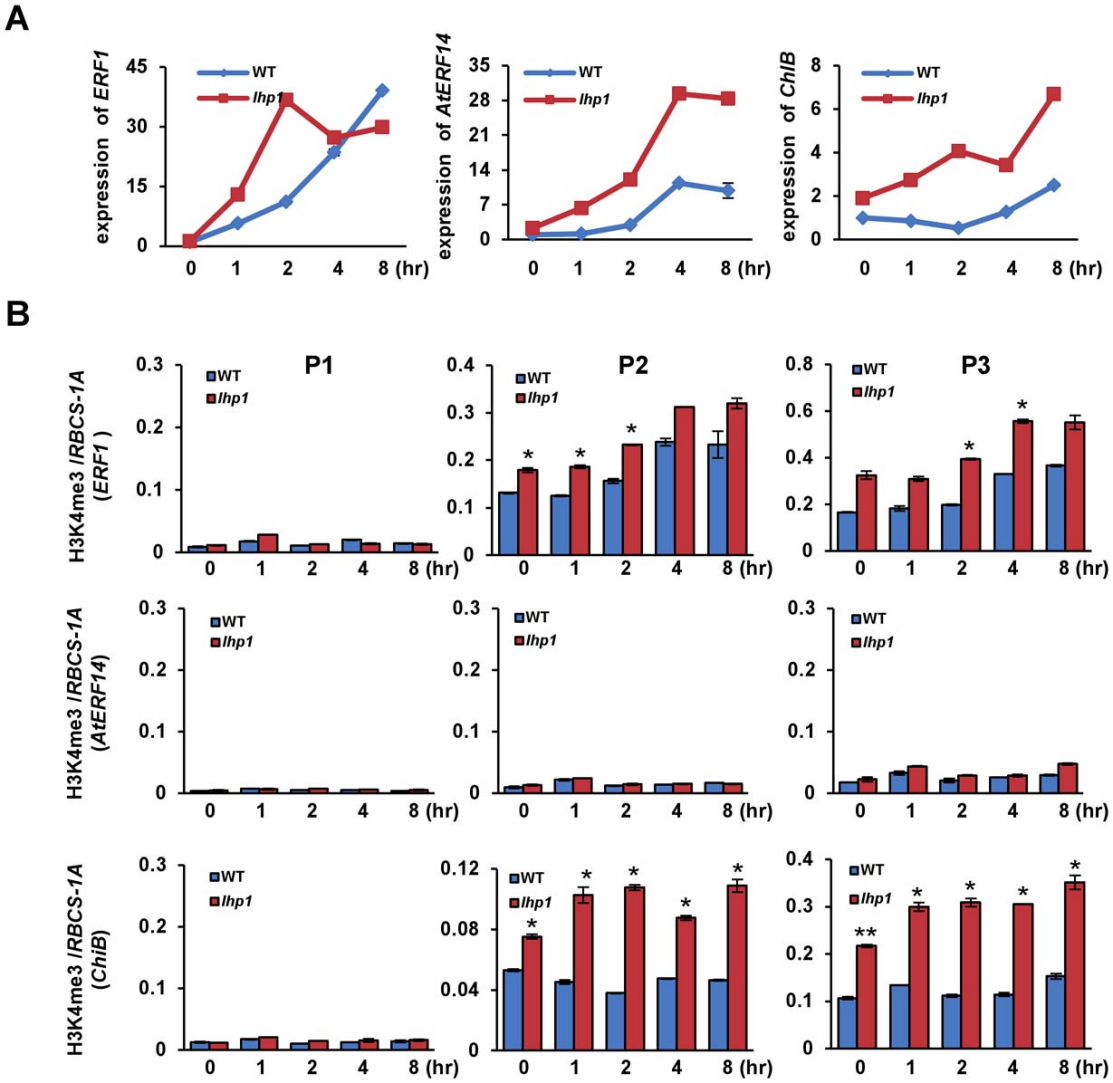
The role of LHP1 on the induction of ethylene responsive genes. (A) Transcript levels of the three genes at different time points during ACC induction in wild type (WT) and *lhp* plants. Relative fold changes were determined by normalization with *ACTIN2* transcripts. (B) H3K4me3 levels on *ChiB*, *ERF1* and *AtERF14* in wild type (WT) *and lhp1* mutants during treatment. The relative enrichments (to *RBCS-1A*) on three regions of the three genes are presented. Bars represent mean values +/−SD from three repeats. Significance of H3K4me3 differences between WT and *lhp1*at different time points was determined by two-tailed Student's t-test, * p<0.05, **p<0.005.

H3K27me3 is recognized and bound by LHP1 that is suggested to be an H3K27me3 effector. To explore the role of LHP1 in rapid gene activation, we analyzed the induction of ethylene responsive genes in the *lhp1* mutant. For *AtERF14* and *ChiB* that displayed high levels of H3K27me3 the induction by ACC was clearly enhanced in the mutant (Fig. 3A), indicating that LHP1 had a repressive function on induction of the two genes. However, we also detected an elevated expression of *ERF1* in *lhp1* (Fig. 3A). Considering that there was a low level of H3K27me3 over *ERF1* we speculated that this might be an indirect effect of increased expression of *AtERF14,* as it has been reported that overexpression of *AtERF14* could lead to increased expression of *ERF1* [15]. In addition, we tested H3K4me3 levels over the target genes in *lhp1* in comparison with the wild type. We found that in *lhp1* H3K4me3 was increased in the 5′ region and the gene body, but not the promoter, of *ERF1* and *ChiB*. The increased H3K4me3 levels may be also a consequence of increased transcription activity of the genes as mentioned before. However, H3K4me3 remained undetectable over *AtERF14* despite the increased expression of this gene in *lhp1* (Fig. 3B). The early induction of 4 of the 5 additional *ERF* genes was found to be enhanced in the *lhp1* mutant. Except TDR1 that had no H3K4me3, the other three genes displayed increased H3K4me3 (Fig. S3).

**Figure 4:**
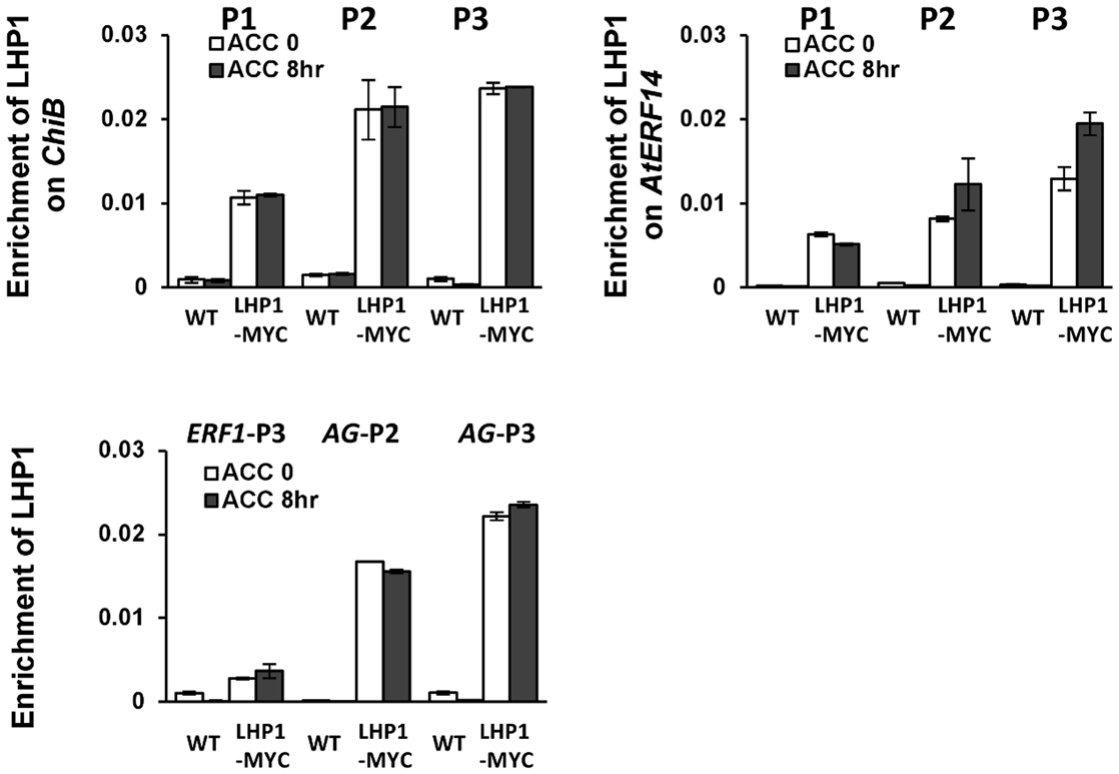
LHP1 binding to different genes before and after ACC treatment. WT and *lhp1* mutants complemented by *LHP1::LHP1-MYC* were used for ChIP analysis with anti-MYC antibodies. The relative enrichments were normalized with input. Bars represent mean values +/−SD from three repeats.

To study whether LHP1 was bound to *ChiB* and *AtERF14*, ChIP analysis of the *lhp1* mutant complemented by *LHP1::LHP1-MYC* was performed by using anti-MYC antibodies[16]. The analysis revealed that LHP1 was associated with *ChiB* and *AtERF14* as well as with *AG*, but not with *ERF1* (Fig. 4). Importantly, ACC treatment did not lead to dissociation of LHP1 from these genes (Fig. 4). Therefore, although the *lhp1* mutation had an effect on the induction of ethylene-induced genes, the presence of the H3K27me3 /LHP1 module on the genes was irresponsive to the inductive signal.

### Requirement of H2A.Z for the induction of *ERF1* and *ChiB*

**Figure 5:**
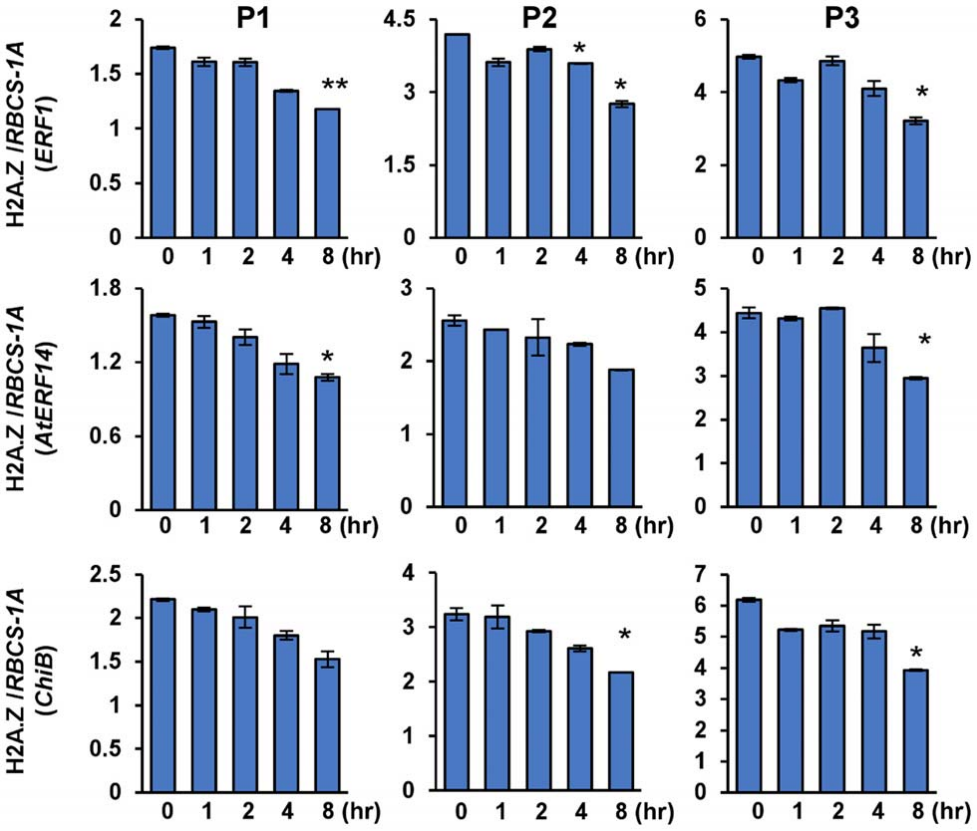
H2A.Z deposition over ethylene responsive genes during ACC induction. H2A.Z-GFP transgenic plants were used for ChIP analysis with GFP antibody. The enrichments on three regions are shown. Bars represent mean values +/−SD from three repeats. Significance of H2A.Z level difference between induced (at different time points) and non induced (0) was determined by two-tailed Student's t-test, * p<0.05, **p<0.005.

In Arabidopsis, H2A.Z has been shown to be involved in multiple responses such as temperature and phosphate starvation [5], [7]. It either activates or represses target genes expression by eviction from nucleosomes occupying around the transcription start site. The presence of H2A.Z was detected over the three ethylene-inducible genes before ACC induction (Fig. 1D). During ACC treatment, no immediate decrease of H2A.Z abundance over ethylene-responsive genes was detected, albeit a slight decrease was observed after induction (Fig. 5; Fig. S2), suggesting that a clear H2A.Z eviction was not required for the initial induction of the genes.

**Figure 6:**
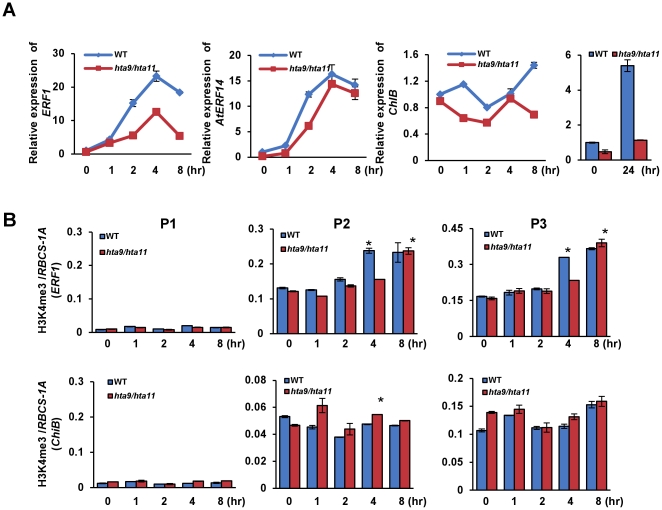
Function of H2A.Z in the ACC induction of *ERF1*, *AtERF14* and *ChiB*. (A) Transcript levels of the three genes at different time points during ACC induction in wild type (WT) and *hta9/hta1* mutants. Relative fold changes were determined by normalization with *ACTIN2* transcripts. (B) H3K4me3 levels on *ERF1* and *ChiB* during ACC induction in WT and *hta9/hta11*. The relative enrichments (to *RBCS-1A*) on three regions are shown. Bars represent mean values +/−SD from three repeats. Significance of H3K4me3 difference between WT and *hta9/hta11*at different time points was determined by two-tailed Student's t-test, * p<0.05, **p<0.005.

In order to study whether H2A.Z was required for the induction, we tested the expression of these genes in *hta9/hta11* double mutants that have a reduced level of H2A.Z [17]. In the mutant the induction of *ERF1* was reduced, while that of *AtERF14* was not clearly affected (Fig. 6A). The effect of the mutations on the induction of *ChiB* was detected after 8 hours, but was more severe after 24 hours (Fig. 6A). These results indicated that H2A.Z was involved in the induction of *ERF1* and *ChiB*. Then we tested H3K4me3 over *ERF1* and *ChiB* during ACC induction in the *hta9/hta11* mutants. Increase of H3K4me3 over *ERF1* observed in wild type was delayed in the mutants, but the basal levels of H3K4me3 over both *ERF1* and *ChiB* were not affected (Fig. 6B). We speculated that H2A.Z had no effect on H3K4me3 and the delayed increase of H3K4me3 over *ERF1* might be a result of decreased transcription activity of the gene in the mutants.

## Discussion

### H3K4me3 serves as a mark of gene transcription activity

Genome-wide analysis in plants indicates that H3K4me3 is associated with actively transcribed genes. But how it affects gene expression remains unclear [1], [18]. Some researchers have proposed that this modification may be recognized and bound by specific proteins which act as effectors to control transcription [3], [19], [20]. But other studies have suggested that H3K4me3 could serve as a memory or a mark of active genes [21]. In this study we tried to find out the role of H3K4me3 during activation of ethylene-inducible genes. Our results indicated that elevated H3K4me3 was not necessary for the ethylene-induced gene activation but may serve as a mark of transcription activity of the genes. First, H3K4me3 was not detected over *AtERF14* and *TDR1* before induction and was not increased after ACC treatment (Fig. 1B, Fig. 2B; Fig. S2). Second, although some increase of H3K4me3 was detected in the 5′ region and/or the gene body of *ERF1, ORA59*, *AtERF1*, *AtERF2* and *AtERF11* after induction, the increase of H3K4me3 lagged behind that of gene activation (Fig. 2B; Fig. S2). Finally, the level of H3K4me3 was associated with that of gene expression in *lhp1* and *hta9/hta11* mutant and wild type plants. For instance, H3K4me3 was increased when the induction of *ERF1* and *ChiB* was enhanced in *lhp1* over these genes (Fig. 3). Conversely, the increase of H3K4me3 was delayed when the induction of the genes was attenuated by the mutation of H2A.Z genes (Fig. 6). Although there was no concomitant increase of H3K4me3 with activation of gene expression, it is not excluded the possibility that the basal level of H3K4me3 over *ERF1* and *ChiB* might contribute to the chromatin commitment of these genes for the induction.

### Repressive function of the H3K27me3/LHP1 module on inducible genes

The observations that H3K27me3 did not change over *AtERF14*, *ChiB* and *TDR1* during ACC induction are consistent with recent results showing that although H3K27me3 on the floral time repressor *FLC* is inversely correlated with transcriptional activity, the abundance of this mark is not diminished in the first 12h following activation of transcription [22]. Analysis of cold-inducible genes has detected H3K27me3 to decline only one to several days after application of the inductive signals [23]. These observations suggest that transcription activation may not involve immediate demethylation of H3K27me3, or the presence of H3K27me3 is not sufficient to impair transcriptional activation during induction. This is supported by a recent result showing that vernalization-mediated induction of *VERNALISATION INSENSITIVE 3* (*VIN3*) does not lead to any decrease of H3K27me3 on the locus 40 days after exposure to cold temperature [24], [25].

The observations that the binding of LHP1 to *AtERF14* and *ChiB* was not affected by ACC induction and that the mutation of *LHP1* led to increased induction of the genes suggest that the H3K27me3 /LHP1 module is required for the repression of the genes and the repressive effect could be lifted by additional events during the induction. The constitutive association of LHP1 with these targets is reminiscent of the data showing that LHP1 remains to be associated with *VIN3* chromatin many days after induction by vernalization [25], indicating that the LHP1 binding in that conditions does not lead to gene silencing. These observations collectively suggest that additional elements associated with the H3K27me3/LHP1 module, which can be inactivated by inductive signals, might be involved in H3K27me3/LHP1–mediated gene silencing.

### Involvement of H2A.Z in the induction of gene expression

Our data showing that deposition of histone variant H2A.Z over the eight ethylene-responsive genes was not evicted after ACC induction are in agreement with the findings that H2A.Z is present in both silent and active *FLC* chromatin (Fig. 5; Fig. S2) [8]. Probably, the presence of H2A.Z may mark these genes for induction, supporting the notion that H2A.Z serves to mark active gene and poise silent genes for reactivation [8], [9]. Recent results have shown that H2A.Z is required for both gene activation and repression in responding to warmer temperature [5]. In contrast to the observations on the ethylene-responsive genes, H2A.Z-containing nucleosomes are found to be lost from both up-regulated and down-regulated genes after an increase of temperature. Therefore, the role of H2A.Z in chromatin structure and in gene activity is complex, which may be dependent on the chromatin context of the gene. In addition, our results showed that mutation of H2A.Z genes had an obvious negative effect on the induction of *ERF1* and *ChiB* but not *AtERF14*. Considering the undetectable level of H3K4me3 over *AtERF14* and moderate levels over *ERF1* and *ChiB* we speculate that H2A.Z may have a coordinated effect with H3K4me3 on the activation of these genes during ACC induction.

Chromatin structure is considered as an important regulator of transcription in addition to transcription factors especially for the developmental genes. In this work we tried to figure out whether chromatin modifications take place during activation of rapidly inducible genes. Our work revealed that histone modifications including H3K4me3 and H3K27me3 and presence of chromatin proteins such as LHP1 and H2A.Z did not display any immediate change upon ACC treatment. However, mutation of LHP1 and H2A.Z genes had an effect on the induction suggesting that basal chromatin structure before induction is important for the induction.

## Materials and Methods

### Plant material and Exogenous ACC treatment

The mutants used in this study are *lhp1* [26], *hta9/hta11* [17], *lhp1* complemented by LHP1::MYC [16] and H2A.Z-GFP transgenic plants [5]. Arabidopsis seeds were surface-sterilized and growth at 22°C with a 16 h light/8 h dark (long day) cycle. Twelve days after germination 50 µM ACC solution was added. The samples were harvested at indicated time points.

### RNA extraction and reverse transcription

Total RNA was extracted from twelve day-old seedling using Trizol (Invitrogen). Four µg total RNA were treated first with 1 unit of DNase I (Promega) and then reverse transcribed in a total volume of 20 µL with 0.5 µg oligo(dT)_15_, 0.75 mM dNTPs, 2.5 mM MgCl2, 1 µl ImProm-II reverse transcriptase (Promega). The resulting products were tested by Real-Time PCR with gene specific primers (Table S1).

### Chromatin Immunoprecipitation

Chromatin Immunoprecipitation (ChIP) experiment was performed most as described in [27]. One gram of 12 day-old seedlings before and after ACC treatment were harvested and crosslinked in 1% formaldehyde under vacuum. Nuclei were then extracted with extraction buffers. Chromatin was fragmented to 200–2000 bp by sonication and ChIP was performed using antibodies: c-Myc (Sigma, M4439), H3K4me3 (Cell Signaling, 9751S) H3K27me3 (Millipore, 07–449) and GFP antibody (Abcam, ab290). The precipitated and input DNAs were then analyzed by real-time PCR with gene specific primer sets (Fig. S1, Table S1). At least three biological repeats were performed for the ChIP experiments.

### Real-Time PCR

Real-time PCR was performed in a total volume of 20 µL with 1.0 µl of the reverse transcription or ChIP products, 0.25 µM primers, and 10 µl SYBR Green Master mix (Roche) on a LightCycler 480 real-time PCR machine (Roche) according to the manufacturer's instructions. All primers were annealed at 60°C and run 45 cycles. The ChIP enrichment for GFP, H3K27me3 and H3K4me3 was quantified by comparing the thresholdcycle (C_t_) of the ChIP samples with that of the input and then normalized with the levels of control genes: 2^(Ct of input-Ct of sample ChIP)^ /2^(Ct of input-Ct of control ChIP)^. The expression level of target genes was normalized with that of *ACTIN*: 2^(Ct of actin- Ct of target)^.

## Supporting Information

Figure S1 **Genes used as controls in this study were not affected by ACC treatment.** The expression of *ACTIN2*, *AGAMOUS* (*AG*) and *RBCS1A* was not affected by ACC treatment. For *ACTIN2*, three biological replication of ACC induction were performed. Data represent average means and the expression before ACC induction was set as 1. The expressions of *AG* and *RBCS-1A* were normalized with that of *ACTIN2*.
(TIF)

Figure S2 **Expression, histone methylation and H2A.Z deposition of five additional ethylene responsive factor (ERF) genes during ACC induction.** RNA levels (A), H3K4me3 (B, C), H3K27me3 (D) and H2A.Z (E) of *ORA59* (At1g066160), *TDR1* (At3g23230), *AtERF1*(At4g17500), *ATERF2*(At4g47220) and *ATERF11*(At1g28370) were measured at the different time points during ACC treatment as indicated. Bars represent mean values +/− SD from three repeats. For ChIP experiments, primers corresponding to the promoter (B) and gene bodies (C-E) were used. Significance of H3K4me3, H3K27me3 and H2A.Z induction compared to that before treatment (0) was determined by two-tailed Student's t-test, * p<0.05, **p<0.005.
(TIF)

Figure S3 **Expression and H3K4me3 of additional ethylene responsive factor (ERF) genes between WT and** ***lhp1*** **during ACC induction.** RNA levels (upper) and H3K4me3 (lower) were measured during ACC treatment. Bars represent mean values +/− SD from three repeats. For ChIP experiments, primers corresponding to the gene bodies were used. Significance of H3K4me3 levels between WT and *lhp1* before and after ACC treatment was determined by two-tailed Student's t-test, *p<0.05, **p<0.005.
(TIF)

Table S1 **Sequences of primers used in this study.**
(DOC)
