# Supplementary figures and images for: The Role of Histone Methylation and H2A.Z Occupancy during Rapid Activation of Ethylene Responsive Genes
Source: PLoS One. 2011 Nov 28;6(11):e28224. doi: 10.1371/journal.pone.0028224 (PMC3225391; doi:10.1371/journal.pone.0028224)

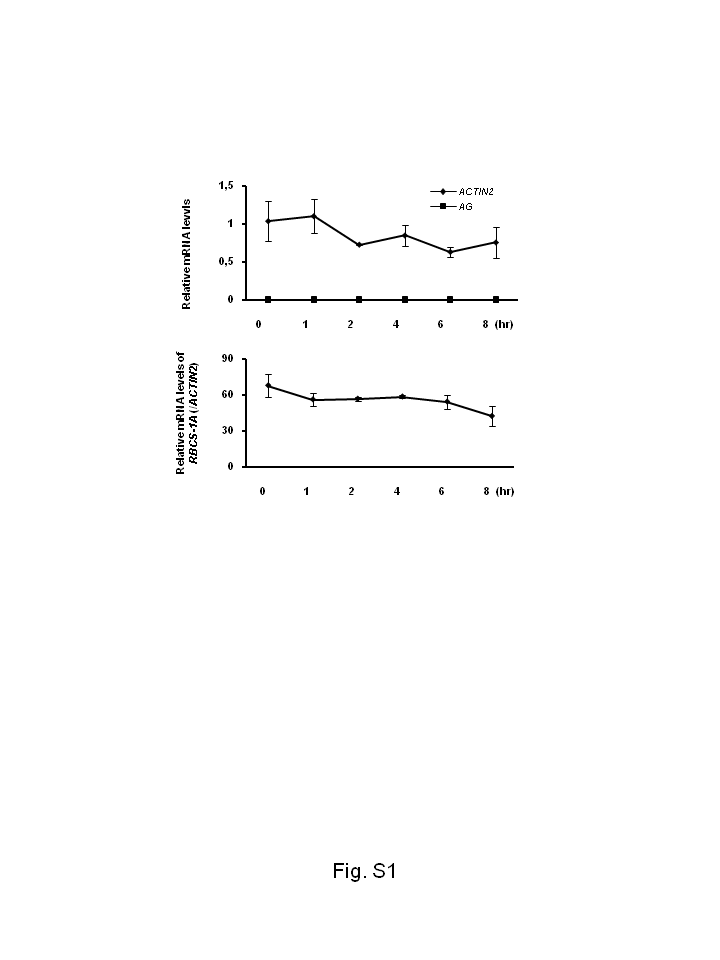

Supplement: Figure S1 — Genes used as controls in this study were not affected by ACC treatment. The expression of ACTIN2, AGAMOUS (AG) and RBCS1A was not affected by ACC treatment. For ACTIN2, three biological replication of ACC induction were performed. Data represent average means and the expression before ACC induction was set as 1. The expressions of AG and RBCS-1A were normalized with that of ACTIN2. (TIF) [file pone.0028224.s001.tif]

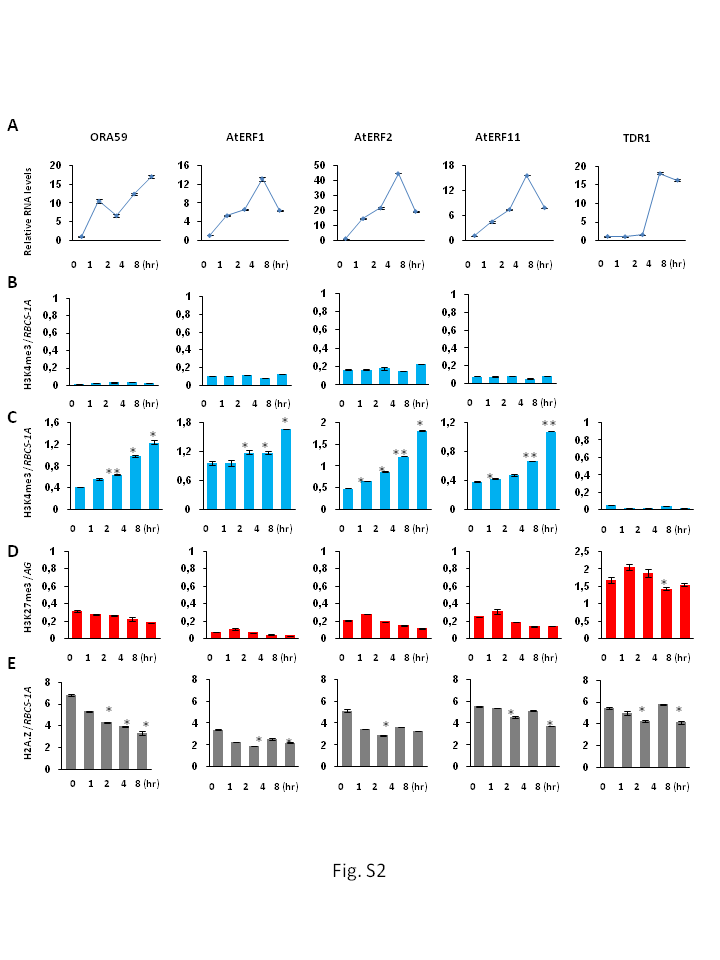

Supplement: Figure S2 — Expression, histone methylation and H2A.Z deposition of five additional ethylene responsive factor (ERF) genes during ACC induction. RNA levels (A), H3K4me3 (B, C), H3K27me3 (D) and H2A.Z (E) of ORA59 (At1g066160), TDR1 (At3g23230), AtERF1(At4g17500), ATERF2(At4g47220) and ATERF11(At1g28370) were measured at the different time points during ACC treatment as indicated. Bars represent mean values +/− SD from three repeats. For ChIP experiments, primers corresponding to the promoter (B) and gene bodies (C-E) were used. Significance of H3K4me3, H3K27me3 and H2A.Z induction compared to that before treatment (0) was determined by two-tailed Student's t-test, * p<0.05, **p<0.005. (TIF) [file pone.0028224.s002.tif]

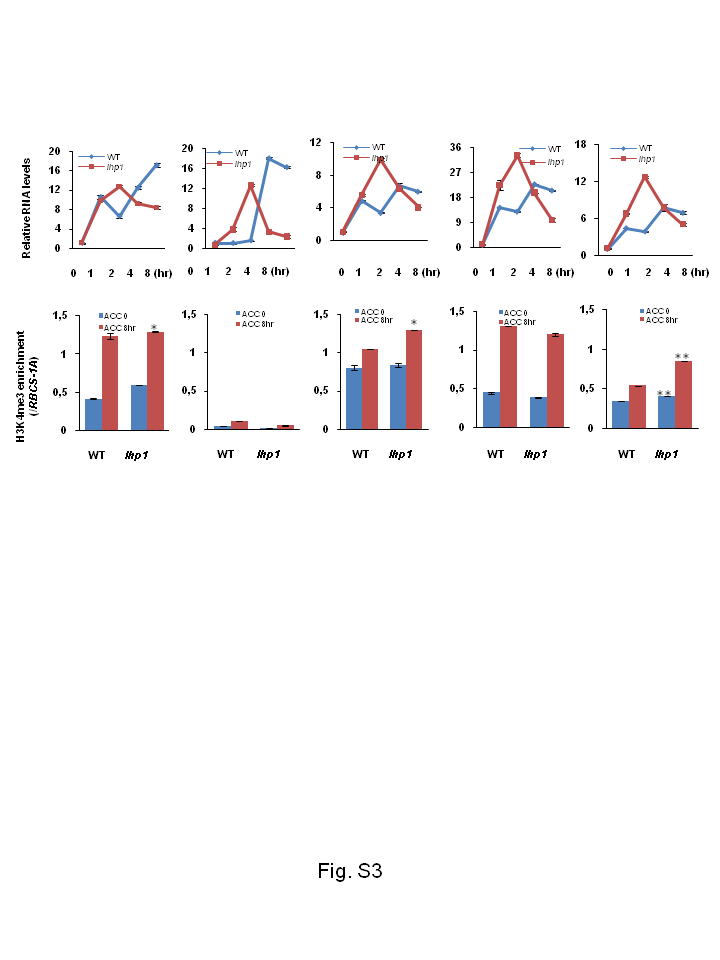

Supplement: Figure S3 — Expression and H3K4me3 of additional ethylene responsive factor (ERF) genes between WT and lhp1 during ACC induction. RNA levels (upper) and H3K4me3 (lower) were measured during ACC treatment. Bars represent mean values +/− SD from three repeats. For ChIP experiments, primers corresponding to the gene bodies were used. Significance of H3K4me3 levels between WT and lhp1 before and after ACC treatment was determined by two-tailed Student's t-test, *p<0.05, **p<0.005. (TIF) [file pone.0028224.s003.tif]
