# Supplementary material for: The Role of Histone Methylation and H2A.Z Occupancy during Rapid Activation of Ethylene Responsive Genes
Source: PLoS One. 2011 Nov 28;6(11):e28224. doi: 10.1371/journal.pone.0028224 (PMC3225391; doi:10.1371/journal.pone.0028224)
Supplement: Table S1 — Sequences of primers used in this study. (DOC) [file pone.0028224.s004.doc]

Table S1. Primers used in this study.

| Primer name | sequence |
| --- | --- |
| ERF1(P1)-F | TGGGTACACCAAAGTAATCCTG |
| ERF1(P1)-R | CGGTAAGGTTTATGAATCATGTACG |
| ERF1(P2)-F | CACTAACGATCCCTAACCGAAA |
| ERF1(P2)-R | TCCGATAGAATATTCCGGTGAG |
| ERF1(P3)-F* | AGGATGGTTGTTCTCCGGTTG |
| ERF1(P3)-R* | ACGGAGCGGTGATCAAAGTCA |
| AtERF14(P1)-F | GGCAACAGCAATGGAATTTT |
| AtERF14(P1)-R | GGTCCAACGAAAGCTTCTGA |
| AtERF14(P2)-F | ACGGACACAAGATGTAGCGTC |
| AtERF14(P2)-R | AGGTTGATGGACCCACTTGTT |
| AtERF14(P3)-F* | GAGCCGCCTATTCAATGAGA |
| AtERF14(P3)-R* | ATCCAAAACGCTATCGTCCA |
| ChiB(P1)-F | CGCCGCCTCATATTCATAA |
| ChiB(P1)-R | CAAGATCACAAGAGTTGCATGA |
| ChiB(P2)-F | TTGTTCCCCATAGCCACACT |
| ChiB(P2)-R | TTCTTTTTGTGGAGGCATGA |
| ChiB(P3)-F* | CTGGCAAACGCTACTACGGAA |
| ChiB(P3)-R* | ACTGCGTCGTTGGCAACAA |
| RBCS1A(ChIP)-F | TGGTCGCTCCTTTCAACGGACTTA |
| RBCS1A(ChIP)-R | CGCCGTTGCTTGTGATGGAAGTAA |
| RBCS1A(RT)-F | CCACCAAGCTTCACCGGTTAA |
| RBCS1A(RT)-R | TCTCGCAAACCGGAAAACA |
| Actin2(RT)-F | CGCTGACCGTATGAGCAAAGA |
| Actin2(RT)-R | GCAAGAATGGAACCACCGATC |
| AG(P2)-F | CAATCGGAGCTAGGAGGAGA |
| AG(P2)-R | AGACGACCACGGCTAGAGAA |
| AG(P3)-F* | AGCTTATGCCACCACCTCAAA |
| AG(P3)-R* | TGTTAGGTTGCAATGCCGC |
| ORA59(ChIP-promoter)-F | AGAGGCCAAAGTGTCGCTAA |
| ORA59(ChIP-promoter)-R | AGAAGTGGGTGAAATGTTCCA |
| ORA59(ChIP-body)-F* | CGGCCGAGATAAGAGACTCAA |
| ORA59(ChIP-body)-R* | GCTGCCTTTCAAAGCGAAAG |
| TDR1-F* | CTTATGACCGAGCAGCCTTT |
| TDR1-R* | AGAAGTTGAACCCGATGACG |
| AtERF1(ChIP-promoter)-F | TCTTCTCCCTGCTCCAACTC |
| AtERF1(ChIP-promoter)-R | GCCGGTTTACCGAAATAAATC |
| AtERF1(ChIP-body)-F* | TAATTCAGGAGAACCCGACCC |
| AtERF1(ChIP-body)-R* | TCACCGTCAATCCCTTATCCA |
| AtERF2(ChIP-promoter)-F | GGAAGAAAGATGCTGATTTGG |
| AtERF2(ChIP-promoter)-R | TGAATTAAGACCTTGGTTTTCG |
| AtERF2(ChIP-body)-F* | TAATTCCGGTGAACCTGACCC |
| AtERF2(ChIP-body)-R* | TCAACTTCCCGTTTTCAGACG |
| AtERF11(ChIP-promoter)-F | TCGCATTTAATCAAAAATAACAGA |
| AtERF11(ChIP-promoter)-R | ATATTGCGTGTGGTGAGGAG |
| AtERF11(ChIP-body)-F* | ACCGTGGAATCATCGTTTCCT |
| AtERF11(ChIP-body)-R* | ATCACCACCGACGAAGAATCC |
